# Examining the social determinants of HIV/AIDS in Madidi village in Bojanala District, North West province

**DOI:** 10.4102/safp.v67i1.6041

**Published:** 2025-03-21

**Authors:** Lebogang E. Lekgema, Phillip Nhlanhla

**Affiliations:** 1 Department of Sociology, Faculty of Human Sciences, University of South Africa, Pretoria, South Africa

**Keywords:** poverty, HIV, AIDS, traditional practices, multiple partners, polygamy, rituals, unemployment

## Abstract

**Background:**

South Africa is experiencing a devastating human immunodeficiency virus (HIV) epidemic, with approximately 7.8 million people living with HIV. International health programs such as the World Health Organization (WHO) and the Centres for Disease Control (CDC) have been assisting citizens in combating the epidemic, but social factors continue to contribute to its spread. The study sought to examine social factors that contribute to the transmission of HIV in Madidi Village, as little is known about this population.

**Methods:**

The study employed a qualitative design and non-probability sampling. Face-to-face, semi-structured, in-depth interviews using a schedule guide were used to obtain data. To capture all of the interviews, a tape recorder was utilised with the permission of the 12 participants with ages ranging from 18 to 49 years.

**Results:**

The study revealed that the majority of participants were aware of HIV transmission and understood that it cannot be cured. However, misconceptions about HIV transmission still exist; for example, one of the participants stated that HIV can be transmitted through Colgate, which has not been scientifically proven to be a transmitter of HIV.

**Conclusion:**

This study shows that people need to be educated more about HIV/AIDS and that the Department of Health officials should play a role in supporting rural areas like Madidi Village to curb the spread of HIV.

**Contribution:**

As women and young girls are the most vulnerable members of the society, they must be enabled to take control of their lives.

## Background

The human immunodeficiency virus (HIV) pandemic is a global issue that has impacted many individuals worldwide. The world has come a long way since the first case of HIV when the mysterious new disease emerged and spread across the globe. Human immunodeficiency virus and acquired immunodeficiency syndrome (AIDS) has claimed many lives and become the leading cause of death in South Africa. However, no cure has been found; hence, prevention of the transmission of HIV remains the main focus. In 2023, over 50 000 people died of HIV-related diseases and an estimated 7.8 million individuals were living with HIV in South Africa.^1,2^ According to the statistics, around 5.7 million people living with HIV (PLWHIV) are undergoing treatment, leaving more than 2 million who should be on ART. Despite the fact that there are preventative strategies and a variety of treatment options, there have been notable new infections, which show that young people between the ages of 15 and 24 years are particularly vulnerable and at risk of acquiring HIV.^3^

According to UNAIDS,^4^ by 2030, South Africa aims to achieve the UN 95-95-95 targets by improving access to treatment and reducing stigma in health facilities, strengthening HIV prevention and ensuring sustainable funding for programmes addressing HIV/AIDS. South Africa is currently at 94-74-67, indicating that 46% of PLWHIV are virally suppressed. According to the HSRC’s 2017 National survey (SABSSMV), the majority of those facing higher HIV risk include young women, drug users, couples who live together and transgender individuals.

The North West province has one of the highest HIV prevalence rates in South Africa, with 12.3% among individuals aged 24–49 years.^5^ The STATS SA Quarterly Labour Force Survey^6^ shows that more than 12 000 people lost their jobs, which led to the rise of unemployment rate of 54.2%. These factors seem to suggest that social factors may contribute to HIV, as people may be involved in risky sexual behaviours such as transactional sex with multiple partners because they lack financial security. Unemployment has shown to be the leading cause of poverty and inequality in South Africa. The researcher saw this as an opportunity to conduct a study in this province, as there is a large number of people infected with HIV and because she wanted to examine the social factors that contribute to the spread of the virus. According to Ratshidze (2021),^7^ North West has the lowest percentatge of people on ARVs and those who are virally suppressed. The main reason for not being able to reach the 95-95-95 target is because of the poor quality of HIV services offered in public sectors. This may affect people’s treatment adherence or cause delays in receiving their medication on time, leading some individuals to default on their treatment.

The research study also presented a description of the research process followed, presented data concerning the methods used when choosing participants suitable for the study and offered theoretical frameworks. The researcher employed a qualitative research approach as it was the most appropriate design for this study. The reason for choosing this approach was that it enabled the researcher to describe and examine social factors that contribute to the spread of HIV/AIDS in Madidi Village in Bojanala District. The researcher conducted a literature review in order to provide a summary of existing information related to the topic. The literature review is followed by a presentation of the theoretical framework relevant to the study. The researcher also described the methods that were used while collecting data. Semi-structured interviews were formulated based on the purpose and objectives of the study; this helped the researcher to focus on the issues that were under investigation, namely social factors contributing to the spread of HIV/AIDS.

## Aim of the study

On the basis of the questions formulated for this research study, the aim of this study was to examine the social determinants that influence the spread of HIV/AIDS in Madidi Village in North West province, which is in Bojanala District. The study also aims to describe the knowledge and lived experiences of people living in the community of Madidi Village regarding social factors that contribute to the spread of HIV/AIDS.

## Research methods

According to Holloway and Gavin,^8^ research methods are procedures, techniques and tools used to gather and analyse data. Maree^9^ added that research method is a process where the researcher collects data in order to analyse, describe and explain the topic at hand. In this study, the methods are discussed under the following headings: research design, data collection and findings.

## Study design

According to Creswell and Creswell,^10^ a research design is a set of formal procedures for collecting, analysing and interpreting data. In this study, a qualitative research approach was deemed suitable for this study, as it allowed the researcher to obtain the voices and commitments of the participants and to explore their experiences and feelings in more depth. According to Mccallum and Howes,^11^ a qualitative study sets out to explore a topic that is infrequently studied in order to understand the experiences and viewpoints of the participants; hence, the researcher employed this research method in order to examine and gain a deeper understanding of social factors that contribute to the spread of HIV/AIDS in Madidi Village.

## Setting

The study was conducted in Madidi Village, which is in Bojanala District, North West province. The population is predominantly composed of Setswana-speaking people as the largest ethnic group in the community. The area comprises 5390 households with an estimated density of 18 300 km^2^.^12^ The area has one clinic providing healthcare services. There is also a skills centre, which offers training and courses for those who cannot afford to go to Universities. The centre also offers counselling services for victims of drugs and alcohol addiction. According to Census,^12^ the area consists of four schools, including three primary schools and a high school.

## Study population and sampling

According to Polit and Beck,^13^ in qualitative research, sampling is a process of choosing a subset of the population to represent the entire population. For this article, non-probability purposive sampling was utilised in order to recruit participants. The target population included both male and female (black African participants), ranging from ages 18 to 49 years. All participants were residents of Madidi Village. The sample criteria included healthcare workers (nurses, a doctor, HIV/AIDS counsellor) as well as unemployed and employed community members. The researcher chose this sampling so that participants can reveal their reflections, personal opinions and experiences that they have about HIV/AIDS and also to unveil meanings associated with selected themes (HIV transmission, unemployment, poverty, lack of supervision, traditional practices, substance abuse, transactional sex, peer pressure, orphanhood and stigma and discrimination). The researcher chose two diverse groups so that they can provide rich data that will assist in answering research questions regarding the phenomenon. This also assisted in increasing the likelihood that the research results would be applicable to a wider audience. Additionally, this also helps in reducing bias.

The inclusion and exclusion criteria that were followed are discussed in the text.

### Inclusion and exclusion criteria

The inclusion criteria for this study included both females and males (nurses, a doctor, HIV/AIDS counsellor, community members who are employed and unemployed) between the ages of 18 and 49 years from Madidi Village in Bojanala District (North West province), who have a general knowledge of HIV/AIDS. The inclusion criteria also consisted of black African participants from Tswana culture who agreed to give consent to participate in the study. Anyone who is not from Madidi Village and is not from Tswana culture is not allowed to participate in the study. Non-community healthcare workers, participants who are below 18 years and above 49 years, were excluded in the study, including anyone with no knowledge of HIV/AIDS.

### Data collection

Data collection is a process of collecting and evaluating information from multiple sources to find answers to the research problem, answer questions, evaluate the outcomes and forecast trends and probabilities.^14^ In this study, before the researcher could conduct the interviews in Madidi Village, which is in Bojanala District, permission was granted from the community leader Mr Sefike who is a ward councillor of Madidi Village ward 3. The researcher also received permission to conduct the study from the Ethics Committee of the College of Human Science at UNISA. Data collection was carried out from 11 November 2023 till 23 November 2023 at Madidi Village in North West province. The researcher developed an interview guide that was used to collect data, and open-ended interview questions were formulated in order to gain information and views of the participants. Open-ended questions were utilised during the interview sessions to enable the participants to speak openly and give detailed descriptions of social factors that contribute to the spread of HIV/AIDS in Madidi Village. Semi-structured interviews were utilised as a data gathering technique in order to gain deeper responses from the participants. A tape recorder was used to record all interviews that took place with the permission from the participants. The researcher used face-to-face interviews as a method of collecting data to give the researcher an opportunity to observe non-verbal behaviours of participants. All transcriptions were anonymised before the analysis. Interviews lasted for an hour and each participant was scheduled for a certain date and time suitable for them.

### Data analysis

According to Dye,^15^ data analysis is a process of collecting, structuring and interpreting data in order to understand what it presents. For this study, analysis of data was conducted using a thematic analysis, following six steps.

Step 1: Becoming familiar with data

The researcher applied Tesch’s analysis method in order to analyse data. This included listening to the audio recordings, transcribing them to verbatim and ensuring validity by comparing the recordings with the transcripts.

Step 2: The researcher generated codes

The researcher organised data systematically with manual coding using Microsoft Word in order to identify codes that could form different themes.

Step 3: The researcher looked for themes

The researcher checked for themes that are essential for providing qualitative research findings. In this study, the researcher established a relationship between themes, subthemes and codes based on the research questions and objectives.

Step 4: The researcher reviewed the themes

After organising the codes into themes, data were then reviewed, modifying themes as needed by separating, combining and refining them.

Step 5: The researcher defined the themes

Subsequently, the researcher read and linked codes with themes, defining and refining them to identify their significance.

Step 6: The researcher wrote a report

Finally, a report was written, incorporating exact extracts from the transcriptions that correlated with themes, objectives, questions and existing literature.

### Ensuring trustworthiness

In order to ensure trustworthiness in the study, the researcher adhered to all the four principles of trustworthiness, namely, credibility, transferability, confirmability and dependability.

### Credibility

To establish credibility, the researcher spent more time in the field engaging with the participants to build trust and to ensure that everything was covered and that data collected were legitimate and consistent. The researcher ensured that data-collection tools were triangulated during interviews by taking field notes and annotating verbal and non-verbal cues.

### Transferability

According to Stalmeijer et al.,^16^ transferability in qualitative research is a quality criterion that measures how applicable a study’s findings are to other contexts, setting or respondents. In this study, the researcher assured transferability by providing a thorough description of research procedures. The researcher used purposive sampling in order to recruit individuals for semi-structured interviews and also to ensure that diverse perspectives are captured.

**TABLE 1:** Demographic information of the participants.

Codes for health workers	Health workers	Codes for community members	Community member
FN1	Female nurse one	FCM1	Female community member one
FN2	Female nurse two	MCM1	Male community member one
FN3	Female nurse three	MCM2	Male community member two
FN4	Female nurse three	MCM3	Male community member three
FC1	Female counsellor one	MCM4	Male community member four
MN1	Male nurse one		
MDR1	Male doctor one		

### Confirmability

To ensure confirmability, adequate time was allocated for data collection in order to ensure that utmost precision was achieved. Field notes and audio recordings were made to serve as reference and support data collected through one-on-one semi-structured interviews. In this study, confirmability was achieved by associating the objectives with interview questions and maintaining proper record keeping. The results of the research could be confirmed through audio recordings, field notes and transcripts.

### Dependability

This study ensured dependability by collecting field notes and paying attention to non-verbal signs throughout the interviews. The researcher then used Tesch’s approach to analyse and evaluate the data acquired.

### Ethical considerations

The researcher obtained permission to conduct this research from the Research and Ethics Committee of the Department of Human Sciences at University of South Africa (reference number 47561599_CREC_2023). All participants who agreed to participate signed consent forms and were informed of the nature of the research study. All data were treated confidentially, and while the researcher knew the participants’ identities, they remained anonymous for the sake of disseminating the research findings. All electronic data were password-protected, while paper material was kept in a cupboard.

## Findings

In this study, the researcher’s population comprised 12 participants, both employed and unemployed healthcare professionals (nurses, doctors and HIV/AIDS counsellors) who have knowledge about HIV/AIDS and are aged 18–49 years.

The following table presents the findings derived from the qualitative data collected during the interviews and the researcher used codes instead of the names of the participants.

## Social factors that contribute to the spread of human immunodeficiency virus/acquired immunodeficiency syndrome

The following themes emerged from the interviews: social factors that contribute to HIV/AIDS and HIV/AIDS transmission.

### Theme 1: Social factors that contribute to human immunodeficiency virus/acquired immunodeficiency syndrome

#### Transactional sex

‘I was very broken, I needed supplements for gym, so I’ve had one and I was very broke and needed the money, yes, so I used that to use me.’ (Participant 3, age 31, male)

Participants were asked if they have ever dated older people for money and MCM3 explained that he once dated a sugar mommy and it was mainly for financial and material gain. He stated that he was broke and needed money for gym supplements. According to Dlamini,^17^ there are times when young men’s motives for dating older women are for financial gain, not love. Therefore, this seems to suggest that young men who are financially unstable prefer older women to take care of them so they can meet their needs. Lehmiller^18^ also indicated that, most of the times, older women date younger boys to feel empowered and to get what they want in a relationship such as sexual needs and otherwise. This seems to suggest that women date younger men to feel satisfied and gain their confidence when sleeping with them. This might also be risky because sometimes you will find that these young boys have girlfriends who are their age mates and who have sexual intercourse with them without using condoms.

#### Poverty

Participants in the study indicated that poverty is also an issue that causes the spread of HIV in the community as it influences sexual behaviour. Other researchers indicated that people may end up migrating from one place to another, which causes families to separate because people need jobs to provide for their families. Some may take long from coming home, which encourages commercial sex and sexual networking where men end up having other partners outside their marriages. This statement was also attested by one of the participants (FN4) as stated further in the text:

‘And then in other cultures you find that … in our case I am in Soshanguve and my husband is working in Mpumalanga having other sexual relationships can contribute mainly to the spread of HIV where you find that the person that you sleep with the woman or man will sleep with another woman without using any form of protection that can cause the spread in HIV. And other issue is that urbanisation, when people move from the areas like Madidi village you move to the city to look for employment and when you get there we know that with our African male, even females nowadays they indulge in extra-marital affairs and they end up sleeping with people that they don’t even know their status.’ (Participant 4, age 42, female)

#### Stigma and discrimination

‘Uhm the stigma uhm its its very bad because uhm a person will fear when I am diagnosed with HIV that I will die, that’s the first thing that they will think and they won’t be free to go to the clinic to take uhm their ARVs, they will end up uhm being very sick because they uhm not taking their medication they default because they don’t want to be seen … they don’t want to be associated with people who are HIV because in in their minds when they think about HIV all they see is death … yes.’ (Participant 2, age 29, female)

This seems to suggest that individuals who stigmatise themselves may experience decreased self-esteem and develop a fear of dying because of a lack of knowledge that they can still live long lives even if they have HIV by adhering to their treatment. Receiving HIV counselling is crucial to avoid such thoughts. Because of lack of knowledge and awareness about HIV as well as negative stereotypes people associate themselves with, there is a need for more education. This seems to suggest that having a feeling of stigma may hinder seeking treatment, and that individuals may prefer not to seek help on time out of fear that their HIV status will be disclosed. Stigma from health professionals should be addressed and dealt with, because health professionals should be a source of support and care to PLWHIV.^19^

#### Unemployment

‘Eeh … one can be: unemployment rate, the high unemployment rate that can be one of the factors and then the other one can be substance abuse and also poverty. I can say that [thinking] uhm polygamy at the other hand and also can be one of the factors. uhm did I mention unemployment?’ (Participant 1, age 39, female)

Participants were questioned to name the social factors that contribute to HIV/AIDS and majority mentioned unemployment, substance abuse and having multiple partners. The participant FN1 indicated that unemployment is the main issue in the community because its rate is so high; she also mentioned other factors such as substance abuse, poverty, polygamy and traditional practices. This seems to suggest that unemployment does affect the society because people are struggling to get jobs even if they are educated or furthered their studies. Unemployment leads to poverty because having no source of income causes people to undergo severe financial hardships, and being homeless and having debts led people to commit crime and engage in risky sexual interactions.

‘Ohk uhm the one social factor that is affecting the community here is unemployment, uhm because uhm a lot of youth here is not working and we are living in a society that has more peer pressure as a younger individual you will see. Uhm things that needs money for you to have them then you will end up involving yourself in prostitution or having sugar daddies, which can lead you to contract this HIV virus, Yes.’ (Participant 2, age 29, female)

Participant FN2 supported the statement made by FN1 by saying that unemployment is a serious factor that affects the community members because a lot of people are struggling to get jobs, especially young people. She further stated that young people are pressured to have material goods that require them to have money. This seems to suggest that youth unemployment is a nationwide problem and it affects young and old people because a majority of people are still complaining about it every year. According to Grigoryeva,^20^ the majority of young people have least seniority experiences and most of the times they are working on short-term contracts. This seems to suggest that unemployment may also be caused by the expectations or requirements the companies have; again, a lot of young people may lack the skills and experiences because they finished their matric or University studies to just qualify for the positions they are applying for, which makes it difficult for them to get employed.

#### Traditional practices

Participants also discussed the traditional practices that cause the spread of HIV/AIDS and one of them mentioned polygamous marriages, circumcision and incision.

‘It’s this one ya circumcision, polygamous marriage, uhm and then this one ya di cutting the skin, bae bitsang kana with razors where the family calls the inyanga and from the last born to the first born including papa and then whoever, they use same blade for cutting them bare baba phatsa ke di traditional beliefs, I don’t know whether they are putting something in them but what they say they say by cutting them they … I don’t know how to put it [laughs] they make you strong against the witches but at the same time they are introducing infections, imagine the youngest child and the eldest child who is sexually active and use a needle that was used for everyone, or papa le mama you don’t know their sexual practices and you take that infection from one person to the other then you end up having the whole family with HIV even the innocent ones.’ (Participant 4, age 42, female)

One of the participants (FN4) added that most of the times in African families, a traditional healer is usually called to perform certain rituals by using a razor blade to cut their skin without sterilising or changing the blade. This ritual may be risky because young children are involved; older people may infect younger ones as they are sexually active, besides trying to cure the illness or performing rituals to protect the family members from witchcraft, which can put them at risk of HIV. Polygamy was also mentioned, and participants in the study stated that men believe polygamy enhances their status and gives them the privilege of having as many children as they want. This indicates that men are ignorant about their health and are not afraid of being infected with sexually transmitted diseases.

#### Lack of parental supervision

‘The other social issues are the … like now if you leave children with people that you don’t even know like the uncles, like in our case you find that young women get pregnant and they live in the same house, so it becomes easy for them to leave their young girls with “bo malome” uncles, trusting that unless will take care of them, but along the way they end up having sexual activities with the … without the parent knowing. Lack of supervision on young girls, we just leave them playing around when they go out they are free and then you won’t even know they are sexually active. And the other sexual factors now in our schools, we don’t have di separate uhm “bare keng kana” toilet facilities and they are not even supervised, our kids end up having sexual activities even at school, knowing that they are at school but they are not supervised, they get HIV at a very tender age.’ (Participant 4, age 42, female)

One of the participants mentioned that lack of supervision may also be a problem that may cause the spread of HIV, especially among young kids if they are left with uncles to take care of them when their parents are at work. Uncles may take advantage by sleeping with them without using any protection. Unsupervised adolescents may particularly be vulnerable to contracting HIV and STDs. The participant also added that unsupervised toilets at school may also cause children to engage in sexual activities knowing that no one can see them. According to Erinosho et al.,^21^ parents who are not available at home leave their children with caretakers or family members when they are at work, thus lacking supervisory control over their children. This seems to suggest that if parents taught their children about how to speak up and report such matters to them, there would be less of such incidents.

### Theme 2: Human immunodeficiency virus transmission

Participants were asked what HIV/AIDS is and how HIV can be transmitted, and one of the participants responded by saying:

#### Substance abuse (sharing of needles)

‘I would say substance abuse is one of the most ignored uhm… contributors towards HIV/AIDS because these guys like I said earlier that they are “Bluetooth” now and then these women they have what we call Alostro, so [pause] so she must have a smoke get a blunt before she sleeps, but she doesn’t have a plan, so she must hustle, she wouldn’t mind whoever she sleeps with, she don’t care about the status, she cares about satisfying her ache for the thing that she’s aching for, so basically it’s very like they … it’s like jumping in a hole knowing this is a hole and it’s 7 metres down, but I don’t care.’ (Participant 3, age 31, male)

A participant in the study explained that substance abuse is one of the most ignored factors in the community that contributes to HIV/AIDS. Drug users are now using what they call Bluetooth, which is a nickname used by addicts to inject themselves with heroin and then withdraw their own blood back into the syringe and inject their friends. This method carries high risk of HIV transmission and other diseases. According to Mahopo,^22^ drug addicts are prone to illness called endocarditis, which is characterised by bacterial infection that attacks heart muscles causing complications such as strokes, kidney failure and fever. This seems to suggest that people end up putting their lives at risks just to feed their cravings. The participant continued to say that girls who are drug addicts are also at risk of contracting HIV because they have what is called *Alostro*, which is a term used for heroin withdrawals, and they end up sleeping with men so that they can buy drugs before going to sleep to just satisfy their cravings. The participant mentioned that people are gambling with their lives because they know the consequences of what they are doing.

#### Unprotected sexual practices

‘My understanding about HIV/AIDS is that it is very dangerous and is that is affecting many people in our community. And how is transmitted is that is through unprotected sex and sharing certain stuff like Colgate and other stuff.’ (Participant 5, age 29, male)

The submission from MCM2 suggests that unprotected sexual contact is the most common way of becoming infected with HIV/AIDS. He also mentioned other possible ways to become infected such as sharing Colgate. The participant brought up the issue of Colgate, which has not been scientifically proven to be a transmitter of HIV. He also mentioned that the virus can spread through other things but he did not say what things. The participant seems to be unsure or unaware of other ways the virus can spread.

#### Orphanhood and mother-to-child transmission

‘And the other kids in the era of HIV before we had “di” treatment we had so many orphans of HIV, you find that the child’s mother was positive and they did not take the treatment or they were afraid to take treatment because of if they take it at any time the person will know that the person is HIV positive, because they have to take treatment a certain time so that they end up not disclosing that their status and not taking treatment. So this young girls and boys when they get in to relationships you find that the child is positive without even them knowing and when they look for antenatal care at the clinic, it is then they will know that they are positive and they will they will know at that time that they are positive and they cannot ask anyone because parents died of HIV and the grannies are at … I don’t know they are fearful they don’t want to tell them, to disclose that their mother passed away because of HIV. So is one of the social issues that will lead to the spread of HIV, fear of disclosing says anything to the kids and then again it is a stigma, the Stigmatisation, they will feel like it’s not allowed, so they keep it as a family secret … mmm.’ (Participant 4, age 42, female)

In the study conducted, the above participant mentioned that there is also high HIV prevalence among orphans who got infected from their mothers during pregnancy or childbirth, without knowing. This may have happened because the mother was afraid of disclosing her HIV status and did not take treatment, which ended up affecting the infant. Orphans who do not know their HIV status are more likely to infect their partners and or even engage in HIV risk behaviours because of losing their source of livelihood or the person who was responsible for taking care of them. This seems to suggest that orphans who do not know their status are more likely to become seriously ill, which could lead to death. Kimani-Murage et al. (2010),^23^ children who are orphans tend to have low levels of food security, which may cause malnutrition, especially because they are not eating healthy because of poverty. On the other hand, the participant explained that sometimes family members who are aware of the status of the child may not want to disclose it because they fear stigma and discrimination.

## Discussion

In this study analysis, the researcher has demonstrated that social factors such as unemployment, traditional practices, poverty, lack of supervision, orphan hood, substance abuse and peer pressure impact the odds of HIV infection even if there are prevention measures that can be considered. The results suggest different pathways for how these social factors impact HIV risks among females and males in society.

### Human immunodeficiency virus transmission

Participants in the study were questioned about their understanding of HIV transmission mechanisms and the majority indicated that it can be transmitted through sharing of sharp tools and injections especially among drug users^24^ as they have developed what we call ‘Bluetooth’. Most drug users are now injecting themselves with drugs and they end up withdrawing their own blood to share with other drug users, which carries a high risk of HIV. According to CDC,^25^ injecting drugs can be a direct route of HIV transmission; if people share needles and sharp tools that are already contaminated, they can infect one another with HIV. This seems to suggest that the majority of the participants were well-informed or are knowledgeable about how one can get HIV and by that they will be able to protect themselves from contracting HIV.

One of the participants indicated that one can get HIV through sharing of Colgate, which has not been scientifically proven. Walker^26^ argued that HIV cannot be transmitted through the use of a toothbrush or toothpaste, as toothpaste can kill the virus before transmission; however, this statement has not been scientifically proven to be true. People should be taught about how HIV can be transmitted to avoid confusion and misinforming others because others may try to experiment whatever they have been told. If effective prevention programmes can be implemented in the community, this can help others to be informed about other routes of HIV transmission.

The article also presented the issue of orphanhood, highlighting the fact that adolescent orphans are likely to have acquired HIV at birth or through breastfeeding, especially if the mothers did not disclose their status to the family or take preventative measures to protect their children from being infected. Geldard and Geldard^27^ additionally stated that some HIV-positive orphans were not lucky to escape HIV because their mothers were not accessing ART during pregnancy. They further stated that the majority of orphans who are infected come from poor family backgrounds and are looking for ways to make money to take care of themselves and gain access to effective medication. As a result, some engage in risky sexual behaviours. One participant mentioned that some individuals may not disclose their HIV status and end up infecting their partners, especially if they do not know their HIV status. The participant further stated that some only discover their status when they visit healthcare facilities for antenatal care with their partners, as their parents or elders may have withheld this information because of fear of stigma.

### Social factors that contribute to human immunodeficiency virus

Questions addressing how social factors contribute to the spread of HIV/AIDS were posed. Our findings indicated that stigma and discrimination contribute to the spread of HIV, as they cause delays in treatment among those living with HIV and those who have not been diagnosed with HIV as they might be afraid to get tested for HIV. This statement is supported by Sherrell,^28^ who stated that people who usually experience stigma and discrimination may not seek treatment and testing out of fear for being judged, which indicates that people may end up defaulting on their treatment and end up dying as the virus might worsen. DeCarlo et al.^29^ further added that this might also increase the chances of people developing low self-esteem. People need to be educated about HIV stigma and also receive post-HIV counselling, which may help to deal with such reactions and psychological distress; this will also help in increasing the number of people taking their treatment on time. Stigma and discrimination should be targeted at many levels involving PLWHIV in the design, implementation and evaluation of stigma campaigns and programmes. People should be taught about HIV transmission, prevention and care, as well as legal rights to access health services.

Our exploratory analyses also found that unemployment, which is linked to poverty, is one of the social factors increasing the chances of being infected with HIV because people end up having multiple partners as they are financially unstable. Our findings also showed that there are high HIV prevalence rates among women who are poor as they may lack control over decision-making when it comes to their health. Limited opportunities were identified as drivers of risky sexual practices, as living in poverty causes food insufficiency, which ends up contributing to HIV.^30^ This shows that unemployment and poverty are associated with HIV because those who are unemployed desperately need money to take care of themselves.

Our findings also identified that traditional practices were also mentioned as social factors of HIV/AIDS, which includes practices such as incision where all family members, including children, are cut using razor blades to insert muthi or herbs, for circumcision and polygamy. Traditional practices can have positive effects, as traditional healers are able to treat certain opportunistic infections, such as childhood diseases. However, some practices may put people at risk of acquiring HIV, particularly when the same razor blades are reused and the traditional healers are exposed to blood from the clients. This shows that people are deeply rooted to their cultures and still prefer consulting traditional healers rather than going to hospitals to seek medical help unless they are seriously ill. Similar findings were reported in the study ‘Clinician-led training to improve personal protective equipment use among traditional healers’, which stated that medical care is not sought early when someone is sick and this might negatively impact the treatment of HIV if they are HIV-positive.^31^ The findings also mentioned that traditional practices such as polygamy and circumcision are the drivers of HIV; for example, people in polygamous marriages have more than one sexual partner,^32^ which is risky especially if others are not faithful. People are likely to acquire STIs, including HIV, especially if no condoms are being used. Women in polygamous marriages may also be exposed to power differences where they have no right to make their own decisions when coming to sexual practices.^33^ In addition, women who lack economic empowerment and education are more likely to experience emotional abuse^34^ and economic dependence compared to those who are independent. This seems to suggest that some women may opt to be in polygamy marriages because they are being taken care of financially, not considering their health.

From our analysis, lack of supervision was also mentioned as a social factor that contributes to the spread of HIV. One participant indicated that some parents may lack the ability to provide guidance or look after their children as they are at work, so they end up leaving them with their uncles who end up taking advantage of them, especially young girls. The vulnerability of young girls to sexual abuse may include exposure to perpetrators of child sexual abuse who target young girls for sexual gratification, including some of the family members.^35^ This shows that children who are not supervised may be at risk of contracting sexually transmitted diseases as well as HIV because older people are already sexually active. Again, the participants indicated that children at schools may also not be safe because toilet facilities are not supervised; this might lead them to engage in sexual practices, which may put them at risk of infecting one another as some are born with HIV. This seems to suggest that children who are not supervised are more likely to engage in sexual activities without condoms, which may also increase the chances of teenage pregnancies. The absence of supervision could deter early sexual experimentation among young children. Studies have shown that a lot of young people learn these behaviours from friends as well as from social media, where they end up wanting to practice them. Jones^36^ mentioned that media have greater influence on sexual behaviours, especially among young people.

Our findings showed that transactional sex is a social factor that contributes to HIV/AIDS, which is driven by relationships in which one partner provides money while the other one provides sex. This increases the chances of one getting infected with HIV, especially if it happens with multiple partners. Others may find it difficult to leave such relationships as it maintains their lifestyles. Such relationships end up being riskier in the sense that the other partner might suggest non-condom use, while the other is unable to negotiate safe sexual practices, especially if they are being paid a lot of money.^37^ In such relationships, you will find that a woman is older than the man,^38^ where she is the one who provides material goods or money in exchange for sex. This seems to suggest that individuals may do anything just to maintain the lifestyles they desire, not considering the consequences, especially regarding their health.

### Limitations

A qualitative research approach was appropriate for this study but led to some limitations. The researcher used one-on-one interviews in qualitative research, which limited participant’s opinions and experiences or honest answers. Instead, they may try to impress the researcher by sharing information that might make them look good in a socially acceptable manner.

### Recommendation

Based on the findings of this study, we recommend that traditional healers should collaborate with healthcare providers to ensure the safety of traditional practices like circumcision, particularly in initiation schools. This partnership would allow for the integration of modern medical knowledge to minimise the risk of disease transmission and complications during these ceremonies. Additionally, healthcare providers can offer training, guidance and resources to traditional healers to enhance sterilisation techniques and infection prevention measures. This collaboration would promote communication and coordination between traditional healers and healthcare providers, ultimately prioritising the health and well-being of initiates. Efforts to prevent HIV/AIDS should prioritise strategies tailored to the cultural and social context, particularly those that empower women. Implementing interventions that empower women is crucial, as certain cultural practices perpetuate oppression and gender inequality, often promoting male dominance. By addressing these cultural norms and providing support and opportunities for women, such interventions can help shift power dynamics and promote gender equality, ultimately contributing to more effective HIV/AIDS prevention efforts. In addition, women in the community should receive education on self-respect and their inherent value, as some may compromise their health and well-being by engaging in risky behaviour, such as unprotected sex for financial gain. Implementing laws^34,39^ that empower women to make their own decisions is crucial in protecting them from being controlled or oppressed by cultural norms that prioritise male dominance.

## Conclusion

The study findings reaffirm that social factors such as unemployment, poverty, lack of supervision, orphanhood, substance abuse, traditional practices, stigma and discrimination and transactional sex predispose individuals in the community, particularly women, to a heightened risk of HIV infection. Changing the power disparities that currently exist in male–female relationships is one of the most serious challenges facing AIDS prevention in South Africa. Only then will women have the ability to protect themselves. Women and young girls need to be empowered so that they may take charge of their lives, as they are the most vulnerable ones in the society.
